# Mechanical Strength Enhancement of Polylactic Acid Hybrid Composites

**DOI:** 10.3390/polym11020349

**Published:** 2019-02-17

**Authors:** Ji-Won Park, Jae-Ho Shin, Gyu-Seong Shim, Kyeng-Bo Sim, Seong-Wook Jang, Hyun-Joong Kim

**Affiliations:** 1 Lab of Adhesion and Bio-Composite, Program in Environmental Materials Science, Department of Forest Science, Seoul National University, Seoul 08826, Korea; roorouny@gmail.com (J.-W.P.); pass2462@snu.ac.kr (J.-H.S.); sks6567@snu.ac.kr (G.-S.S.); skb181@snu.ac.kr (K.-B.S.); jangsw0202@snu.ac.kr (S.-W.J.); 2 Research Institute of Agriculture and Life Sciences, Seoul National University, Seoul 08826, Korea

**Keywords:** microencapsulated phase change material (MPCM), polylactic acid (PLA), toughening, endothermic effect, kenaf fiber, hybrid composites

## Abstract

In recent years, there has been an increasing need for materials that are environmentally friendly and have functional properties. Polylactic acid (PLA) is a biomass-based polymer, which has attracted research attention as an eco-friendly material. Various studies have been conducted on functionality imparting and performance improvement to extend the field of application of PLA. Particularly, research on natural fiber-reinforced composites have been conducted to simultaneously improve their environmental friendliness and mechanical strength. Research interest in hybrid composites using two or more fillers to realize multiple functions are also increasing. Phase change materials (PCMs) absorb and emit energy through phase transition and can be used as a micro encapsulated structure. In this study, we fabricated hybrid composites using microcapsulated PCM (MPCM) and the natural fibrous filler, kenaf. We aimed to fabricate a composite material with improved endothermic characteristics, mechanical performance, and environmental friendliness. We analyzed the endothermic properties of MPCM and the structural characteristics of two fillers and finally produced an eco-friendly composite material. The PCM and kenaf contents were varied to observe changes in the performance of the hybrid composites. The endothermic properties were determined through differential scanning calorimetry, whereas changes in the physical properties of the hybrid composite were determined by measuring the mechanical properties.

## 1. Introduction

Polymer-based packaging materials are increasingly utilized because they are easy to process, can be molded into a variety of designs, and are lightweight [1,2,3]. The demand for materials with special functionality is increasing owing to their use in diverse applications such as ultraviolet (UV) absorption, special heating, and thermal shielding [4]. Among them, the heating function is one of the core functions required in biomedical and cosmetic industries, as the contents can be safely preserved from thermal deformation [5,6]. In addition, polymer-based packaging materials are developed as eco-friendly materials to mitigate environmental problems caused by waste generated during use [7,8].

Polylactic acid (PLA) is a thermoplastic polymer with a linear aliphatic polyester structure, which can be obtained by polymerizing monomers from renewable resources such as corn and potato starch [9,10]. PLA is biodegradable, environmentally friendly, and can be used in various applications, such as bio-plastic. Therefore, the use of these biodegradable materials can reduce the burden on container waste and the cost of environmental preservation. Various studies have been conducted on imparting functionality and performance improvement to extend the field of application of PLA. Particularly, research on natural fiber-reinforced composites have been carried out to simultaneously improve the environmental friendliness and mechanical strength [11,12].

Phase change material (PCM) refers to thermal storage material used for controlling temperature changes [13]. PCMs are substances that accumulate or store heat through a type of physical change process from solid to liquid state, from liquid to solid state, from liquid to gaseous state, and from gaseous to liquid state. As the external temperature increases, the PCM reaches its own melting point. The material then changes phase from solid to liquid state and absorbs a certain amount of heat, known as melting enthalpy. The temperature of the material remains constant despite the application of heat. These properties are suitable for energy conservation and are increasingly utilized in the energy sector [14,15].

A fibrous, reinforced material with natural fibers has a low aspect ratio compared to conventional fibrous materials. Although its mechanical reinforcement performance is poor, its lightweight structure and low cost of the material makes it an economical option [16,17]. It also has the strength needed to maximize the utilization of biomass as well as to improve the durability of materials. PCM, on the other hand, is typically composed of a micro-shell type in which a shell protects the core material. Therefore, if the two materials are used simultaneously as fillers, other structural reinforcement effects can be achieved in one material. A composite material that utilizes such a heterostructure system is known as a hybrid composite material [18]. 

Result predictions in hybrid composites are difficult, as they are expected to have different effects for different fillers. The direction of the analysis changes according to the degree of interaction between the filler and the matrix. In hybrid composites research based on natural fibers, synergistic effects are expressed using the interaction of the two fillers with the matrix [18]. In the study of ceramic and natural-fiber hybrid composite, only the change in strength of one filler has been confirmed [19].

In this study, we investigated changes in the mechanical properties when fillers with different structures are dispersed in a PLA matrix. The study aimed to verify the feasibility of developing eco-friendly composites with improved endothermic characteristic and mechanical performance. In particular, we investigated the effect of toughening when using core-shell particles and the possibility of improving the performance. We analyzed the characteristics of PCM and kenaf fiber and investigated the feasibility of producing a new type of biocomposite material with multiple functions. 

## 2. Experimental

### 2.1. Materials

In this study, the polymer matrix used in green composites was PLA, which was obtained from NatureWorks LLC (Minnetonka, MN, USA). The PLA was provided in granular form with an average diameter of 81 μm and density of 1.24 g/cm^3^. Its melt flow index (MFI), glass transition temperature (*T_g_*), and melting temperature (*T_m_*) were 7 g/10 min (210 °C/2160 g), 57.3 ± 0.6 °C, and 146 ± 6 °C. Microencapsulated PCMs (MPCMs) were obtained from J&C Microchem, Inc. (Anseong-si, Korea) and the core consisted of paraffin wax, whereas the shell was composed of melamine formaldehyde resin. The kenaf fibers were donated by Sutongsang Company (Gyeongju-si, Korea) which was manufactured as 200 meshes using a pulverizing machine. The fibers were laid out in plastic bags after drying at 80 °C for 24 h to remove excess moisture.

### 2.2. Preparation of MPCMs/Kenaf Fiber/PLA Hybrid Composites

The PLA was dried at 80 °C for 12 h and stored in polyethylene bags. The dried PLA pellets were compounded with 10 and 20 phr (parts per hundred resin) of MPCMs and 10, 30, and 50 phr of kenaf fiber using a laboratory sized twin-screw extruder (BA-19 in Bautek, Pocheon, Korea) The barrel of the extruder was divided into eight zones and the barrel temperature zones of the extruder when extruding the materials were 185/195/200/200/200/195/165/140. The speed of the screws was maintained at 200 rpm. After extruding the materials, the extruded strand was cooled in a water bath and pelletized using a pelletizer (Bautek, Korea). The zone temperature control played a key role since the dispersion of the PCM and the kenaf fiber had to be induced. Table 1 presents the mixing conditions of the hybrid composite. In general, the higher the temperature, the better the flowability of PLA and the better the dispersion. However, when cellulose is the main component of natural fiber, it is carbonized when exposed for a long time at a temperature higher than 180 °C. Byproducts from carbonization should be minimized as they affect the color and physical properties of the composite. Therefore, the composite material should be made by varying the temperature and time conditions that minimize the carbonization of kenaf [19].

### 2.3. Characterization 

#### 2.3.1. Scanning Electron Microscopy (SEM)

Field emission scanning electron microscopy (FE-SEM, SUPRA 55VP, Carl Zeiss, Oberkochen, Germany) was performed at acceleration voltage of 10 kV to observe the morphology of the MPCMs. Before the measurement was carried out, the sample was pre-coated with a homogeneous platinum layer (purity, 99.99 %) by ion sputtering to eliminate electron charging.

#### 2.3.2. Differential Scanning Calorimetry (DSC)

Differential scanning calorimetry (DSC) was performed using a DSC Q200 system (TA Instruments, Chicago, IL, USA) with an RCS 90 refrigerator cooler to determine the glass transition temperature (*T_g_*), crystallization temperature (*T_c_*), and melting temperature (*T_m_*) of the MPCMs and composites. Approximately 5 mg of the sample was loaded in a T_zero_ aluminum pan and high-purity nitrogen gas was used as purge gas at a flow rate of 50.0 mL/min. The samples were scanned from 0 to 150 °C at a heating rate of 5 °C/min. 

#### 2.3.3. Tensile and Flexural Strengths

The tensile and flexural strengths of the specimens were measured using a universal testing machine (AllroundLine Z010, 2000N load cell, Zwick, Ulm, Germany) according to the ASTM D 638–10 and ASTM D 790–10 standard test methods. A cross speed of 5 mm/min was used during the measurement and the mechanical properties were analyzed at room temperature. Six specimens were measured to calculate the margin of error.

#### 2.3.4. Izod Impact Test

The Unnotched Izod Impact Strength tests were conducted at room temperature. Each value obtained represents the average for five samples.

## 3. Results and Discussion

**Figure 1:**
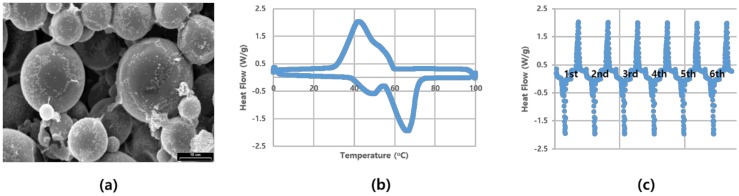
Basic characteristics of microencapsulated phase change material (MPCM): (**a**) SEM image (×2000); (**b**) endothermic/exothermic reaction (bottom—elevated temperature, above—decreased temperature) with DSC; (**c**) cycle test with DSC.

The basic characteristics of the MPCM used in this study were analyzed. The SEM image and thermal properties of the PCM microcell used in this study is shown in Figure 1. The material was partially composed of small particles, mostly in the range of 10–20 μm. DSC was used to identify the endothermic part of MPCM. Furthermore, a cycle test was conducted to determine the stability of MPCM, as it exhibits different thermal characteristics during the heating and cooling process. In this study, we focused on the characteristics of temperature rise. 

**Figure 2:**
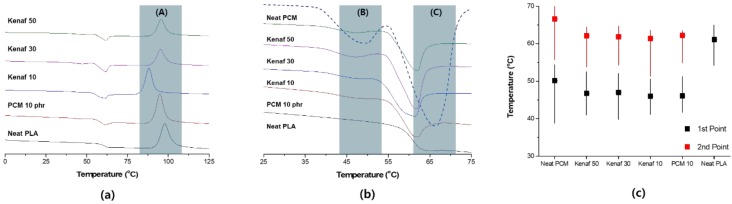
Analysis of heat absorption characteristics of PLA hybrid composites with phase change material (PCM) 10 phr: (**a**) Entire temperature range showing the crystallization section (A); (**b**) expansion of the heat absorption section showing the primary endothermic section (B) and the secondary endothermic section (C); (**c**) endothermic temperature of each sample.

Figure 2 shows the results of the DSC analysis of hybrid composites with a PCM content of 10 phr. When a composite material is formed, the thermal properties of the material are changed by the interaction of the matrix and filler. In this study, the inherent endothermic characteristics of PCM should be fully reflected. Three specific temperature changes can be confirmed by DSC measurement. Zone (A) is the crystallization zone of PLA. The crystallization temperature of PLA is 89.7 °C; in this study, the crystallization temperature was determined as 87.1 °C when only PCM was used and 79.4, 87.8, and 88.4 °C when 10, 30, and 50 phr of kenaf were used, respectively. Thus, the use of PCM resulted in some reduction in the crystallization temperature. This is because the fine particles of PCM serve as a nucleus of PLA crystallization. In this case, when 10 phr of kenaf was added, the crystallization temperature decreased sharply. Small amounts of fibers and PCM particles can improve the mobility of PLA and accelerate crystallization in this process. The particles may also serve as a starting point for some crystallization. However, as the content of kenaf increases, the crystallization temperature increases. As the ratio of fibers increases, the formation of the crystal structure inside the polymer is limited, which affects the increase in the crystallization temperature. Zone (B) is the primary endothermic section formed by PCM; the primary endothermic interval is determined by the change in paraffin wax, which constitutes the PCM. The primary endothermic section of the PCM is 49.1 °C. Such an endothermic section cannot be confirmed at neat PLA. In this study, the primary endothermic section was formed in the range of 47–49 °C when PCM was utilized; this endothermic section also occurred when kenaf was used. Zone (C) is the core endothermic phase by PCM. PCM exhibited the largest endothermic characteristic at 66 °C. The core endothermic phase showed a characteristic that appears at the *T_g_* of PLA, which was formed at 61 °C. However, in the hybrid system, the maximum endothermic characteristic occurred at 62 °C. This was determined to be influenced by the phase change of PLA. Figure 2c shows that the change in the endotherm is not affected by kenaf fiber. The PCM/PLA composites have a higher first endothermic section and a lower second endothermic section compared to neat PCM. The reason the temperature of the first endothermic section is higher is that heat conduction is delayed inside the composite material. On the other hand, it can be interpreted that the second endothermic section changes together with the *T_g_* of the PLA.

**Figure 3:**
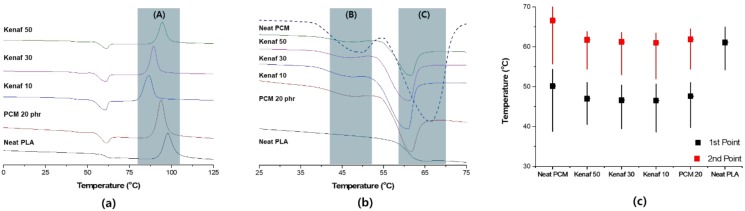
Analysis of heat absorption characteristics of PLA hybrid composites with PCM 20 phr: (**a**) Entire temperature showing the crystallization section (A); (**b**) expansion of the heat absorption section showing the primary endothermic section (B) and the secondary endothermic section (C); (**c**) endothermic temperature of each sample.

The results of the DSC analysis of hybrid composites with a PCM content of 20 phr are shown in Figure 3. As the content of PCM increased, the main peak became more pronounced. The crystallization temperatures were 84.8 °C for only PCM 20 phr, and 77.6 °C, 82.2 °C, and 86.5 °C for 10 phr, 30 phr, and 50 phr, respectively. When kenaf was used, the endothermic temperature was partially reduced, however, it tended to increase minimally as the content increased. Zones (B) and (C) are similar to those obtained in PCM 10 phr test group. It was observed that the endothermic curve was more pronounced owing to the increase in the content of PCM. 

**Figure 4:**
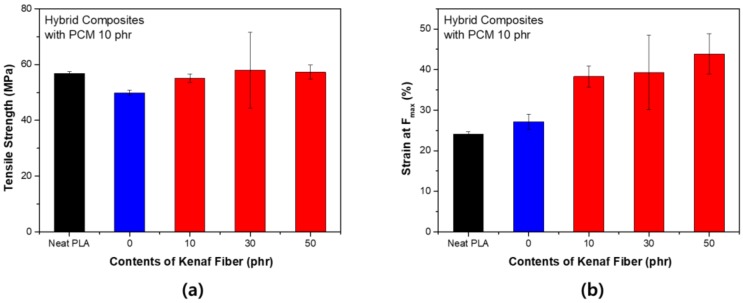
Tensile strength of PLA hybrid composites with PCM 10 phr: (**a**) Maximum stress (F_max_); (**b**) strain at F_max_.

**Figure 5:**
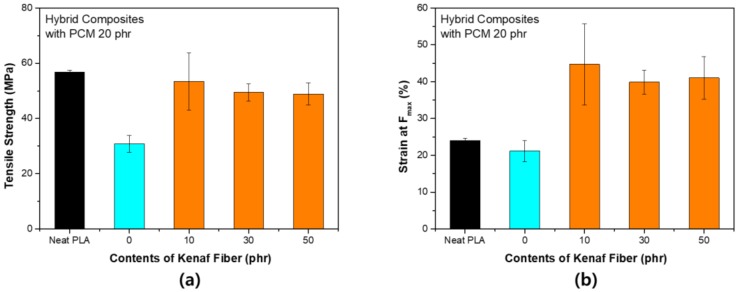
Tensile strength of PLA hybrid composites with PCM 20 phr: (**a**) Maximum stress (F_max_); (**b**) strain at F_max_.

Figure 4 shows the change in the tensile strength according to the content of kenaf with PCM 10 phr. When PCM was used, the tensile strength was slightly reduced, however, the reduction in strength was restored by adding kenaf. The notable result was that the tensile elongation increased as the strength was maintained. In general, the use of fibers such as kenaf increases the stiffness of the composite and thus tends to decrease tensile elongation. This tendency is considered to be due to the structural characteristics of PCM. Unlike kenaf, PCM is free of strain. These deformation characteristics complement the section where the fracture is formed during the tensile process. As a result, resistance to fracture tends to improve, which is known as the toughening effect. In general, PLA is a brittle polymer, and such properties must be improved when the material is used. This trend has the same effect even when the content of PCM increases. As shown in Figure 5, the tensile strength decreased significantly when the content of PCM was increased to 20 phr. However, tensile strength recovery by kenaf fiber occurred in a similar process. The tensile elongation was larger at small contents of PCM. PCM was distributed throughout and served to compensate for the increase in stiffness due to kenaf. It can be concluded that PCM, which is a spherical particle, and kenaf, which is a linear particle, complement each other. The use of linear fillers increases the modulus of elasticity, but leads to brittleness. When a spherical filler is used, the impact absorption properties are strengthened, whereas the tensile strength is weakened. However, the two materials used in this study tend to overcome the disadvantages of using each material individually through a synergistic effect [20].

**Figure 6:**
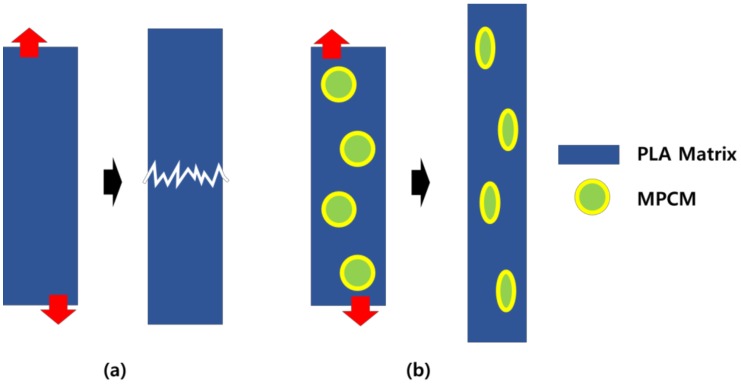
Estimation of the toughening effect according to use of MPCM: (**a**) Neat PLA; (**b**) PLA with MPCM.

Figure 6 shows a graphical representation of the changing tensile failure phenomena when MPCM was used. The improved tensile properties of MPCM were due to the increased degree of freedom, which changed internally through the use of MPCM. A neat PLA is a material with Poisson's ratio of 0.36 [21] and is vulnerable to deformation. However, if the MPCM is dispersed internally, it compresses, making it relatively advantageous to vary the tensile strength. The tensile strength characteristics change depending on the structural characteristics [22]. When hybrid filler is used, the spherical filler is placed between the fibrous filler and matrix and the fluidity of the polymer is enhanced. Therefore, the hybrid filler exhibits a completely different characteristic compared to when it is used individually. The use of two fillers tends to increase brittleness, however, when used together, a synergistic toughening effect can be expected [23,24].

**Figure 7:**
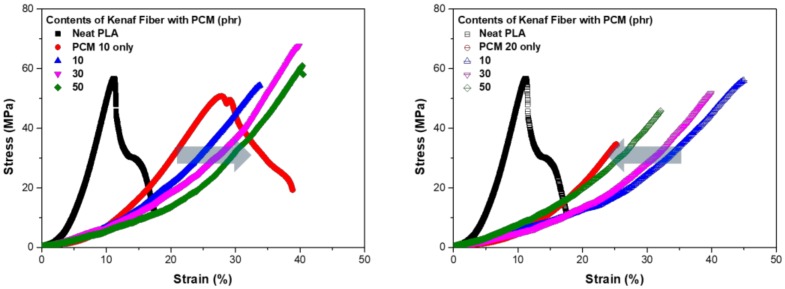
S–S curve from tensile strength test: (**a**) PCM 10 phr; (**b**) PCM 20 phr.

Figure 7 shows the S–S curve from the tensile strength test of each sample. In Figure 7a,b, the strength decreased when PCM was used, whereas the graph tended to move upward when kenaf was used. At a PCM content of 10 phr, the graph shifted to the right as the kenaf content increased, whereas at a PCM content of 20 phr, the graph shifted to the left as the kenaf content increased. The shift in the graph indicates that the characteristics of the material were changing. At a small content of PCM, a hybrid effect occurred between PCM and kenaf fibers, whereas when the PCM content increased, it can be considered that a negative effect occurred in the spatial arrangement. PCM, which spatially occupies a large area, inhibits the dispersion of kenaf fibers so that a negative result can occur. At a PCM content of 10 phr, the tensile strength increased slightly when kenaf was added, whereas the strength tended to decrease slightly at a PCM content of 20 phr. The two trends can be interpreted to be due to the increase in the spatial ratio of the PCM and kenaf fibers in the composite material. The distribution of the matrix is important for the composite material to exhibit sufficient mechanical strength. As the filler content increased, the dispersion and bonding of the filler were adversely affected.

**Figure 8:**
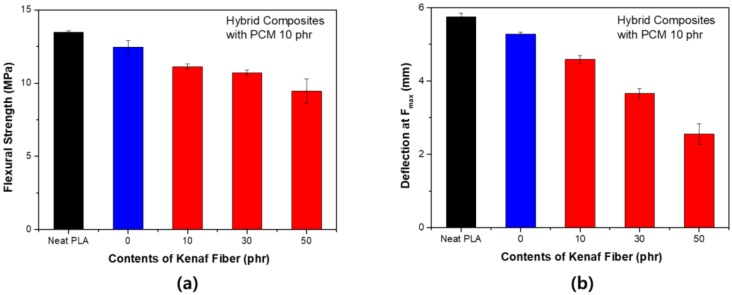
Flexural strength of PLA hybrid composites with PCM 10 phr: (**a**) Maximum stress (F_max_); (**b**) deflection at F_max_.

**Figure 9:**
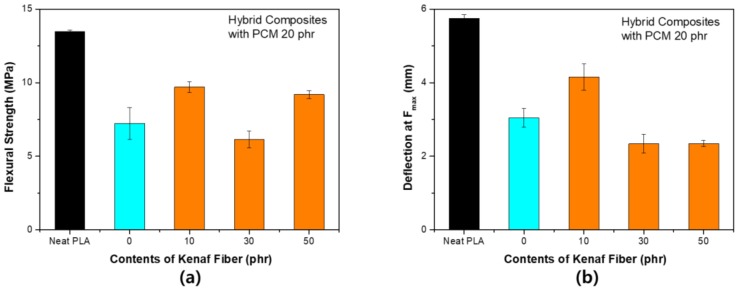
Flexural strength of PLA hybrid composites with PCM 20 phr: (**a**) Maximum stress (F_max_); (**b**) deflection at F_max_.

Figure 8 shows the change in the flexural strength according to the content of kenaf for PCM 10 phr. The flexural strength tended to be quite different from the tensile strength. As the content of kenaf increased, both the flexural strength and the maximum load deflection tended to decrease. The fibers did not play a major role in reinforcing the mechanical strength during the bending process, since the surface of the kenaf fiber was not subjected to any surface treatment. In addition, PCM has a relatively flexible shell. The shell compensates for fracture during deformation in the tensile process, however, in the bending process, it reduces the flexural strength according to the fracture due to compression and weak interfacial bonding characteristics. When the content of PCM increased to 20 phr, a larger decrease occurred. However, as shown in Figure 9, the flexural strength seemed to be partially restored by the partial reinforcement effect of the fibers. There was no overall tendency for PCM 20 phr. This is because the bending strength decreased significantly when only the PCM was used. It is considered that even if kenaf fibers were used, the strength could not be supplemented.

**Figure 10:**
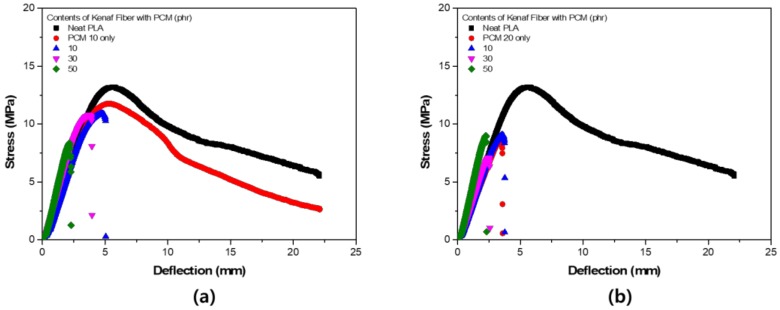
S–S curve of flexural strength test: (**a**) PCM 10 phr; (**b**) PCM 20 phr.

Figure 10 shows the S–S curve obtained from the flexural strength test of each specimen. As the filler was used differently compared to the tensile strength, the slope of the graph increased, and the maximum strength tended to decrease. The S–S curves between samples were not significantly different under PCM 20 phr conditions. These results highlighted the irregularity of the results in Figure 9. The fracture shape at the flexural strength showed a completely different tendency compared to that of the tensile strength. In the process of using the composite filler, the destruction tended to proceed straight after the yield point. The elastic modulus at the flexural strength can be expressed by the following equation [25]:

(1) Flexural modulus=FL34wdh3

where *L* is the support span, *w* and *h* are the width and height of the beam, respectively, and *d* is the deflection due to the load *F* applied in the middle of the beam. In this case, *L*/(*wh*) is constant. Therefore, the elastic modulus is proportional to the strength and inversely proportional to the deflection.

(2) Flexural modulus∝Fd

**Figure 11:**
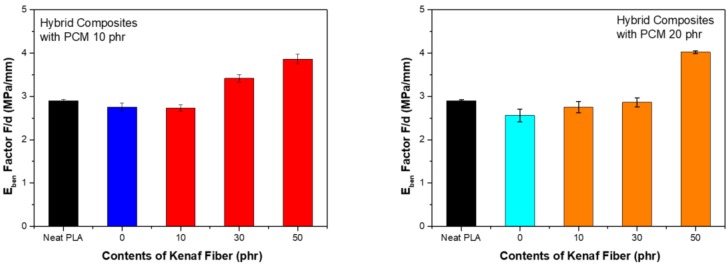
Scale factor of flexural modulus: (**a**) PCM 10 phr; (**b**) PCM 20 phr.

Figure 11 shows the scale factor of the flexural modulus. The utilization of PCM served to reduce the scale factor. This means that PCM did not play a strong binding role in compression and tensioning of the material. On the other hand, when kenaf was used, the scale factor tended to increase as the content increased. The increase in the elasticity factor was considered to be due to the fiber-reinforcing effect. The strong elastic force of the fiber was reflected in the interaction with the fiber, but the bond with the fiber deteriorated, leading to the destruction. These characteristics were considered to be the result of the short fiber-reinforced structure.

**Figure 12:**
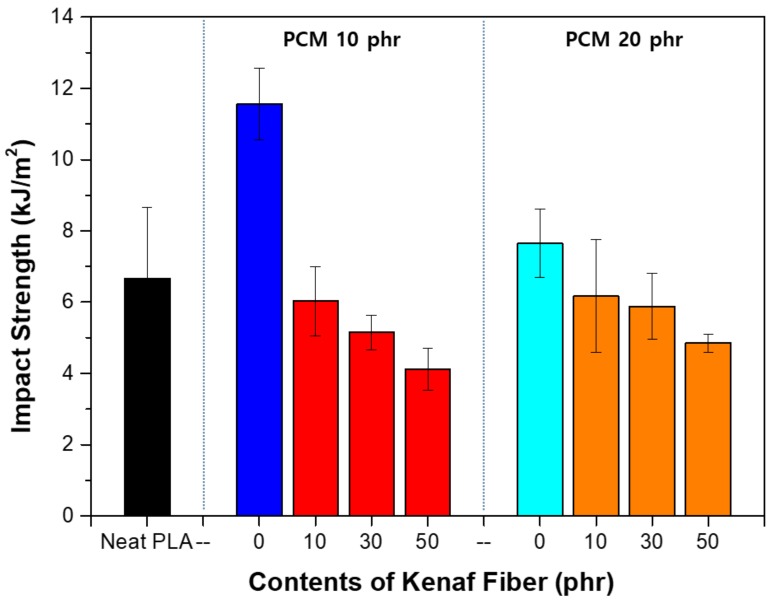
Impact strength of PLA hybrid composites.

Figure 12 shows the impact strength evaluation results of each specimen. When PCM was utilized, the impact strength tended to increase, whereas when kenaf was used, the impact strength tended to decrease. When PCM alone was used, the impact strength greatly increased when 10 phr is used, but when the PCM was used at 20 phr, the strength tended to decrease again. The impact strength was improved when the spherical filler is used, but the strength tended to reduce when the specific amount was exceeded. As the amount of filler increases, the bond between the fillers also strengthens. The optimum content depends on the size and shape of the filler [26]. In the previous tensile strength test, the tensile elongation increased, but the tensile strength did not change significantly. Overall, the modulus of elasticity tended to decrease, which indicates that the material itself was becoming soft. It can be deduced that the impact strength tended to decrease as the physical properties of the material weakened. In general, the fiber reinforced composite material tended to exhibit increased impact strength depending on the fiber content. However, in this case, it can be expected that the fiber acted as a crack initiation point for reducing the impact strength owing to the weak bonding force between the fiber and the matrix. Thus, the increase in the fiber content had a negative effect.

**Figure 13:**
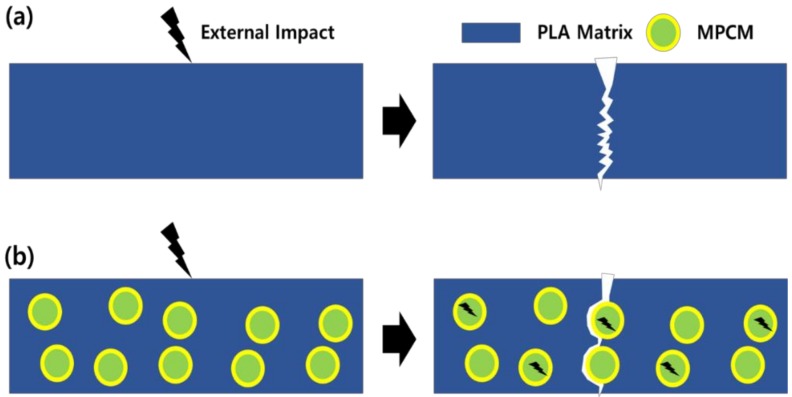
Toughening effect according to use of MPCM under external effect: (**a**) Neat PLA; (**b**) PLA with MPCM.

Figure 13 shows the toughening effect process of MPCM. The toughening effect of MPCM was confirmed again by the impact strength evaluation. When an external impact is applied, it is possible to change the fracture pathway and at the same time absorb the impact so that excellent toughening effect can be achieved. The impact absorption characteristics tended to decrease with the content because the porosity increased inside the composite due to an increase in the bonds between the particles present in Figure 13.

## 4. Conclusions

Hybrid composites fabricated using MPCM and kenaf fibers reflect the characteristics of the fillers. The endothermic characteristics tended to increase as the content of PCM increased. The use of kenaf fibers reinforced both the tensile strength and elongation. However, both fillers had a negative influence on the flexural strength. The toughening effect of the micro-shell was verified by evaluating the impact strength. The results showed that the weaker interfacial bonding force had a negative effect on the impact strength. By combining the fillers with two different structures and functions, we identified areas where complementary effects were realized and where they were not. Therefore, the findings of this study will be useful in developing eco-friendly composites with improved functions in the future.

## Figures and Tables

**Table 1:** PLA (polylactic acid) hybrid composite mixing conditions (weight ratio).

Materials	Matrix	Filler	
	PLA	MPCM	Kenaf Fiber
HC.PLA 1-0	100	10	0
HC.PLA 1-1			10
HC.PLA 1-3			30
HC.PLA 1-5			50
HC.PLA 2-0		20	0
HC.PLA 2-1			10
HC.PLA 2-3			30
HC.PLA 2-5			50
